# Hurricane Helene, One Year Later: Lived Experience

**DOI:** 10.13023/jah.0704.09

**Published:** 2025-12-01

**Authors:** Geraldine A. Miller

**Affiliations:** Appalachian State University

**Keywords:** Appalachia, Ashe County, Hurricane Helene

## Abstract

Ashe County is located in the northwestern corner of North Carolina (NC), bordered by Virginia and Tennessee. The county is isolated, mountainous, and historically has lacked good highway access. Hurricane Helene, a category 4 hurricane at landfall, struck Western NC on September 27, 2024, resulting in flooding and wind damage that destroyed local communities in terms of land, homes, bridges, water, electricity, etc. with a damage of $59.6 billion.^6^ One year later, the recovery from the hurricane continues draining individuals and communities of their time, energy, and money. Geri Miller reflects on her hurricane impact experiences a year later. She is a psychologist that responded to 9/11 in New York as an American Red Cross (ARC) Disaster Mental Health Counselor (DMHC). At 70 years old, she lives in Ashe County along the New River.

Ashe County is located in the northwestern corner of North Carolina (NC), bordered by Virginia (VA) and Tennessee (TN). It is a rural area that has been described as having Christmas trees^1^ and farmland (e.g., beef cattle).^3^ It has a relatively small population of 26,431 residents as of July 1, 2024.^6^ It was once called “The Lost Province,” a name derived from the county’s historic isolation and limited transportation access that oriented trade toward VA rather than the rest of NC. This has resulted in the county being considered a separate state.^5^,^2^ It is possible that this separate state may have:

“…helped shape a fierce independence and a strong resiliency that enable folks to persevere even through bone-chilling winters, economic challenges and feeling like the ‘state stepchild.’ Maybe our relatively isolated territory and mountainous terrain, resulting in at least decades of a shortage of good highways in and out of Ashe, accounted for a feeling of separateness and a mindset of not being totally validated and included by many of the state powers that be.” 4

At times, it seems as though we are bypassed and neglected.^4^

On September 27, 2024, Hurricane Helene, a category 4 hurricane at landfall, originated in the western Caribbean Sea and struck western NC after sweeping through the southeastern coast of the United States. Though Helene had been downgraded to a tropical storm by the time it struck NC,^8^ the flooding and wind destroyed local communities, damaged homes, and disrupted water and electricity, causing an estimated $59.6 billion in statewide damage.^6^ Additionally, OSBM noted that state-owned facilities in Ashe County alone sustained more than $10 million in damage.^7^ The damage impacted the communication systems (e.g., internet, phone) as well as transportation and other infrastructures.

I call Hurricane Helene “North Carolina’s 9/11” because of my 9/11 DMHS work in New York – the only difference being the breadth of the destruction. This article, an attempt to have the “Hollow Voices” from Ashe County heard through my voice, preserves the history of these powerful stories.

Three story areas are important to highlight: *trauma triggers*; *state of powerlessness*; and *living in the shadows*.

One *trauma trigger* is a heavy rainstorm. For example, September 27, 2024, was a Thursday; this year, the Thursday before the actual one-year anniversary, a heavy rainstorm occurred in my area. I drove home wondering if I would make it safely (as my car was hydroplaning) and wondering too if other drivers who had driven during the first night of the hurricane were also scared of the rain, wind, and darkness that spelled a familiar danger. Over the following days, I heard stories of individuals who had similar responses to mine. In spite of these fears, we continue to be brave and live through the hurricane triggers.

When Helene came, the storm’s intensity hit quickly and tossed us into a *state of powerlessness*. A year later, we experience powerlessness reactions through feelings of grief, gratitude, and compassion. Grief and gratitude are intertwined: grief for what was lost and can never be regained (e.g., lives, land), but simultaneous gratitude for being alive. We celebrate as bridges, roads, and other items are recreated with our and others’ resources of time, energy, and money. While some individuals and organizations continue to provide us with these resources, others have moved on, possibly unaware of our ongoing recovery needs. As a result, our commitment to family, community, and tradition has deepened and expanded. I take this lack of acknowledgement with the knowledge that we, as people, increasingly cannot bear the suffering of others without taking action; we worry about the poor, the homeless, and the marginalized people (i.e., anyone who has limited resources and options). In this continuing ocean of need, we face hard choices as to how much time, energy, and money each of us has to give and to whom.

As we continue to grieve the loss of individuals important to us, we also live with the damage to our social fabric. For example, I live outside of a small rural town. We lost our post office, where we crossed paths and shared the stories of our daily lives. Now, we drive quite a distance to reach the closest post office. However, in the summer of 2026, when the new post office is opened, this social fabric will be restored, thanks to the allocation of resources to aid us in our recovery.

Additionally, our sense of humor about our powerlessness, past and present, has assisted us in our recovery. As we live life on life’s terms and navigate recovery, we insist on enjoying life through humor. While no one was aware of the severity of the incoming storm, our response to hearing current weather predictions has long been: “If you want to know what the weather’s doing, go outside.” Such humorous dismissal of the weather has been enhanced by the experience of Helene.

While there is hope and faith that we will fully recover, there is a deep sadness lingering for those who do not have such luck. For example, a lot of media attention was and is legitimately given to Asheville, NC and its surrounding areas, such as Chimney Rock, NC. Those mountain towns in other areas such as Ashe County continue to “*live in the shadows*” of areas that have received media attention; we hunger to have our stories heard.

In summary, our love sustains us in the hollows. We love our families, communities, and traditions. We celebrate when events are held for the first time since the hurricane, bridges are rebuilt, and anything that holds value for us is restored. We are committed to finding a recovery solution to whatever problem presents itself. When the world gives us time, energy, and money in our recovery process, we are grateful, but when it seems to forget about our ongoing recovery, we still take time to be kind and helpful to each other. This unwavering collective spirit, woven from mutual care and shared experience, gives voice to our enduring resilience and hope. Our stories are important, and publications such as this one keep our stories alive and provide attention to our ongoing recovery.
